# Exploring Trends in Neuromonitoring Use in a General Pediatric ICU: The Need for Standardized Guidance

**DOI:** 10.3390/children9070934

**Published:** 2022-06-22

**Authors:** Nathan Chang, Lindsey Rasmussen

**Affiliations:** Pediatric Critical Care Medicine and Neurocritical Care, Lucile Packard Children’s Hospital Stanford, Palo Alto, CA 94304, USA; lkrasmus@stanford.edu

**Keywords:** neurophysiological monitoring, intensive care units, pediatric, neurology, critical care, hospital mortality

## Abstract

Neuromonitoring has become more standardized in adult neurocritical care, but the utility of different neuromonitoring modalities in children remains debated. We aimed to describe the use of neuromonitoring in critically ill children with and without primary neurological diseases. We conducted a retrospective review of patients admitted to a 32-bed, non-cardiac PICU during a 12-month period. Neuro-imaging, electroencephalogram (EEG), cerebral oximetry (NIRS), automated pupillometry, transcranial doppler (TCD), intracranial pressure (ICP) monitoring, brain tissue oxygenation (PbtO_2_), primary diagnosis, and outcome were extracted. Neuromonitoring use by primary diagnosis and associations with outcome were observed. Of 1946 patients, 420 received neuro-imaging or neuromonitoring. Primary non-neurological diagnoses most frequently receiving neuromonitoring were respiratory, hematologic/oncologic, gastrointestinal/liver, and infectious/inflammatory. The most frequently used technologies among non-neurological diagnoses were neuro-imaging, EEG, pupillometry, and NIRS. In the multivariate analysis, pupillometry use was associated with mortality, and EEG, NIRS, and neuro-imaging use were associated with disability. Frequencies of TCD and PbtO2 use were too small for analysis. Neuromonitoring is prevalent among various diagnoses in the PICU, without clear benefit on outcomes when used in an ad hoc fashion. We need standard guidance around who, when, and how neuromonitoring should be applied to improve the care of critically ill children.

## 1. Introduction

Neurologic diseases are major causes of morbidity and mortality in children [1]. Single and multicenter studies suggest that acute neurologic insults can comprise around 20% of pediatric intensive care unit (PICU) admissions [2,3,4,5]. Diseases such as traumatic brain injury, status epilepticus, hypoxic ischemic injury, stroke, and meningitis/encephalitis are associated with higher rates of death and disability when compared to the general PICU population [3,4,5,6]. In this setting, pediatric neurocritical care (PNCC) services and neuromonitoring technologies are emerging as resources with vast ranging potential benefits to children in the PICU [7,8,9].

Frameworks for clinical practice using multimodal monitoring and physiologic goal-directed treatment are becoming more prevalent in adult brain injury and might improve neurologic outcomes [10]. Some clinical guidance and thresholds exist for neuromonitoring technologies in adult populations, such as cerebral near-infrared spectroscopy (NIRS), transcranial doppler (TCD), and electroencephalogram (EEG) in post-cardiac arrest and extracorporeal membrane oxygenation (ECMO) [11,12,13]. Similarly, neuromonitoring has noted benefits in anesthesia for certain intraoperative settings [14]. In most pediatric centers, though, the availability and utilization of different neuromonitoring technologies lack equivalent consensus, and the impacts of neuromonitoring on outcome are likewise unknown [9,15].

Forms of neuromonitoring in children are vast, spanning from noninvasive technologies, such as NIRS, sensory evoked potentials, and TCD, to invasive monitoring, such as ICP monitors or cerebral microdialysis. However, in a recent survey of North American pediatric centers, only intracranial pressure (ICP) monitoring and EEG were shared by all responding institutions [9]. While some studies suggest a feasible benefit to singular modes of neuromonitoring or multimodal monitoring, this benefit has yet to be proven in children [16,17,18]. Furthermore, specificities around who performs neuromonitoring, how it is interpreted, which patients should receive monitoring, and when monitoring should be applied remain unknown. This presents a gap in our ability to use neuromonitoring in a fashion most likely to yield clinical benefit.

Exploring current clinical practice by quantifying neurocritical care resource use can help inform the development of subspecialty service models, clinical education, and standards that are currently lacking in children. To this end, we describe our institution’s experience with the use of neuromonitoring technologies in general PICU patients and identify associations between neuromonitoring use and patient outcomes.

## 2. Materials and Methods

We conducted a retrospective chart review of all hospital admissions to our institution that involved a PICU stay between 1 January 2019 and 31 December 2019. Our PICU is a 32-bed closed unit, independent from the cardiovascular ICU. Our institution is a quaternary care children’s hospital that operates a formal PNCC program, without dedicated specialty Neuro-ICU beds implemented at the time of this study. Our study was approved by our center’s institutional review board. Data were obtained through the third-party Virtual Pediatric Systems (VPS) database and the electronic health record. The numbers of patients who received neuro-imaging, EEG, TCD, intracranial pressure (ICP) monitoring, NIRS, automated pupillometry, and brain tissue oxygenation monitoring (PbtO_2_) while in PICU were extracted. Demographic data, discharge diagnosis, probability of death determined from the highest admission Pediatric Risk of Mortality III score (PRISM III POD), ECMO, hospital length of stay, new tracheostomy or enteral feeding tube requirement on hospital discharge, and discharge disposition were collected.

The primary diagnosis was determined by a proprietary STAR diagnosis provided by VPS based on ICD-10 coding in the medical record, confirmed by discharge diagnosis listed on discharge summaries, and assigned to larger diagnostic categories by an organ system. Diagnostic categories were neurologic/neurosurgical (neuro), respiratory/airway (respiratory), gastrointestinal/liver (GI), renal/genitourinary (renal), hematologic/oncologic/lymphatic (heme/onc), infectious/inflammatory/autoimmune (inflammatory), toxic/metabolic/endocrine (toxic/metabolic), cardiovascular, and other. New significant disability was defined as a new tracheostomy requirement, new enteral feeding tube requirement, discharge to inpatient rehabilitation, or first-time discharge to a skilled nursing facility.

Bedside neuromonitoring was separated into two categories: Non-invasive and invasive. Non-invasive bedside neuromonitoring included EEG, NIRS, automated pupillometry, and TCD. Invasive bedside neuromonitoring included ICP and PbtO2 monitoring. Neuro-imaging included brain or spine MRI, CT scan, or head ultrasound. During the study period, institutional guidelines were only established for NIRS and EEG monitoring in patients on ECMO. Outside of this subset of patients, our institution did not have clinical standards for routine use of neuromonitoring technologies, and thus, neuromonitoring was employed at the provider or nurse’s discretion.

The statistical package for the social sciences (SPSS) software was used for the analysis. Univariate analyses were conducted by two-tailed Mann–Whitney U Test for continuous variables and Pearson’s Chi-square Test for categorical variables or, for samples with frequency less than five, a two-tailed Fisher’s Exact Test. A *p*-value < 0.05 was used for significance. Variables with a positive association with death or disability and a *p*-value < 0.05 in the univariate analysis were included in the multivariate logistic regression; variables with fewer than 10 outcome occurrences were not included in regression. ECMO and diagnostic categories with adequate frequency of outcome were included in regression, because of potential confounding with neuromonitoring use.

## 3. Results

A total of 1946 unique patient hospital visits with PICU stays were observed. Primary neurological diagnoses comprised of 542 (27.9%) patients. Patients with primary neurological diagnoses were on average older, with higher predicted mortality, and experienced a higher rate of death and disability when compared to patients with primary non-neurological diagnoses (Table 1). Individual data is available in the supplementary materials.

In total, 420 (21.6%) of all PICU patients received at least one form of neuro-imaging or neuromonitoring. Of these 420 patients, 331 (78.8%) had a primary neurological diagnosis. These diagnoses included traumatic brain injury (57), brain tumor (54), seizures/status epilepticus (46), hydrocephalus/increased ICP (35), ischemic or hemorrhagic stroke (29), epilepsy surgery (25), meningitis/encephalitis (23), out-of-hospital cardiac arrest or drowning (14), spinal cord injury or lesion (12), other postoperative neurosurgery (30), and other medical neurological diagnoses (6). Furthermore, of the 420 patients who received neuro-imaging or neuromonitoring, 89 (21.2%) had a primary non-neurological diagnosis. These diagnoses were viral bronchiolitis/pneumonia (14), leukemia/lymphoma (11), septic shock (8), liver failure/transplantation (6), heart failure (5), drug overdose (4), and varied other diagnoses (41).

**Figure 1:**
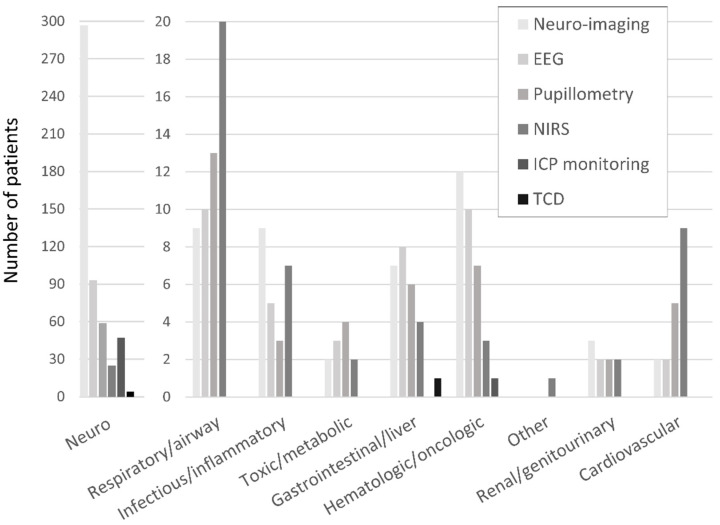
Neuro-imaging and neuromonitoring use by primary diagnostic category.

**Figure 2:**
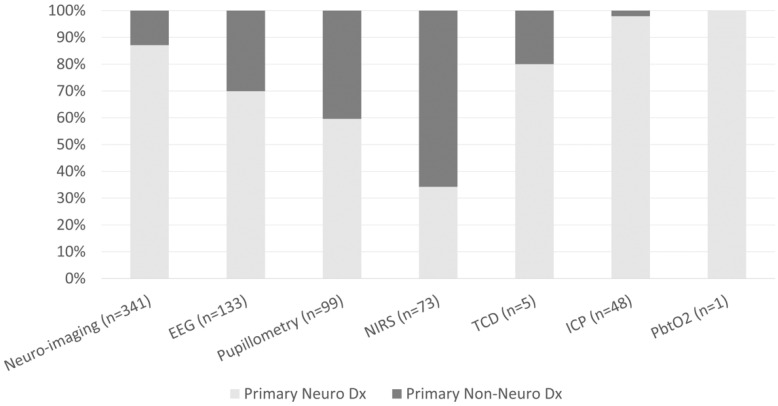
Percentage of patients who received neuro-imaging or neuromonitoring by primary neurological vs non-neurological diagnoses.

The frequencies of neuro-imaging and neuromonitoring use across non-neurological diagnostic categories are listed in Figure 1. A majority of noninvasive and invasive bedside neuromonitoring modalities were allocated to children with primary neurological diseases, with the exception of NIRS (Figure 2). Regarding bedside neuromonitoring modalities, 134 patients received one mode of neuromonitoring, 47 patients received two modes, 34 patients received three modes, six patients received four modes, and one patient received five modes.

Regarding covariates, younger age, neuro diagnoses, and ECMO were associated with death or disability on the univariate analysis with enough frequency to be included in the multivariate analysis (Table 2). Due to a low frequency of outcomes, most diagnostic categories, the use of TCD, and the use of PbtO_2_ monitoring were not included in the multivariate analysis (Table 3).

### 3.1. Non-Invasive Bedside Neuromonitoring

**Figure 3:**
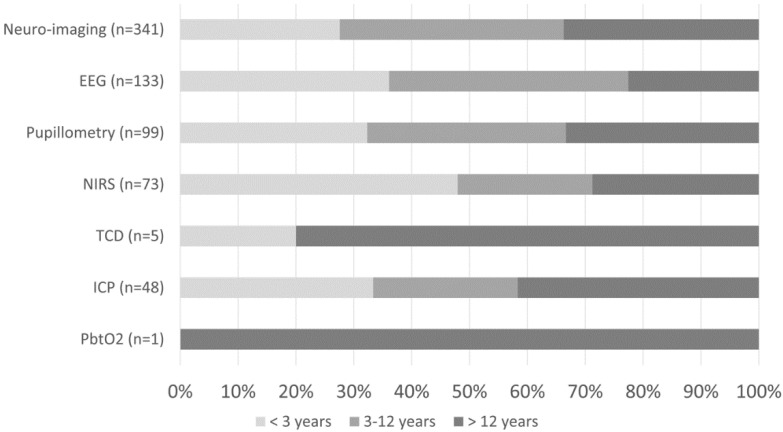
Percentage of neuro-imaging and neuromonitoring use by age group.

At least one mode of non-invasive bedside neuromonitoring, including EEG, NIRS, automated pupillometry, or TCD, was employed in 156 (8%) of all patients. Primary non-neurological diagnoses represented 65.8% of all NIRS use, 40.4% of pupillometry use, and 30.1% of EEG use (Figure 2). Among non-neurological diagnoses, NIRS was most often used in respiratory, cardiovascular, and inflammatory diseases; pupillometry and EEG were most often used in respiratory, heme/onc, and GI diseases. Transcranial doppler was obtained in one patient with GI disease (Figure 1). Regarding age, EEG, pupillometry, and NIRS use appeared generally evenly distributed across age groups, while TCD was used mostly in older patients (Figure 3). Near 20% of patients who received EEG, 34% who received pupillometry, and 31% who received NIRS died (Table 2). On the univariate analysis, each of these modalities were associated with in-hospital mortality (Table 2). On the multivariate analysis, adjusted for covariates and severity of illness, only automated pupillometry was associated with mortality; EEG and NIRS were associated with disability (Table 3).

### 3.2. Invasive Bedside Neuromonitoring

At least one mode of invasive bedside monitoring, including ICP or PbtO_2_ monitoring, was used in 48 (2.5%) of patients. Intracranial pressure monitoring was used largely in patients with primary neurological diagnoses (Figure 2). Regarding ICP monitoring use among non-neurological diagnoses, one patient with heme/onc disease received ICP monitoring (Figure 1). ICP monitoring was generally evenly distributed across age groups (Figure 3). Only 6% of patients who received ICP monitoring died (Table 2). Intracranial pressure monitoring was not significantly associated with death or new disability at discharge in the multivariate regression (Table 3). The least frequently used mode of bedside neuromonitoring was PbtO_2_ monitoring, which was only used in one adolescent patient. Because it was only used in one patient, PbtO_2_ monitoring use was not analyzed for associations with outcome.

### 3.3. Neuro-Imaging

Neuro-imaging was frequently used in all PICU patients and was obtained in 341 (17.5%) patients. Of these patients who received neuro-imaging, 297 (87.1%) carried a primary neuro diagnosis (Figure 2). Regarding non-neuro diagnoses, neuro-imaging was most often obtained in patients with heme/onc, respiratory, and inflammatory diseases (Figure 1). As a percentage of use by age, 27.6% of patients who received neuro-imaging were under three-years-old, 38.7% were between three- and 12-years-old, and 33.7% were over 12-years-old (Figure 3). Near 8% of patients who received neuro-imaging died (Table 2). In the univariate analysis, neuro-imaging was associated with both in-hospital mortality and new disability (Table 2). In the multivariate analysis, adjusted for covariates and severity of illness, neuro-imaging was only associated with disability (Table 3).

## 4. Discussion

We found that near 28% of patients admitted to the PICU had primary neurological diagnoses, higher than some previous reports [1,2,3,4,5,8]. Overall rates of death and disability for primary neuro patients were higher than their non-neuro counterparts, consistent with existing literature [3,4,5]. As a single-center study, our results are unique to the medical and surgical services our institution provides, but our findings further support evidence of a large presence of neurocritical care patients in the PICU. We report considerable neuromonitoring use in patients without primary neurologic diagnoses, as well, suggesting a broader potential benefit of increased attention to neurocritical care resources throughout pediatric critical care [1,2,3,4,5]. Given the positive effects of general protocol adherence on outcomes that have been reported in neurocritical care, this may reflect an opportunity to improve neurologic outcomes in all critically ill patients, at least in part, by standardizing guidance around neuromonitoring [19].

Our study expands on a recent multi-center survey about neuromonitoring use in pediatric centers in North America and highlights the need for future research to answer the question of for whom neuromonitoring should be optimally applied [9]. Whether due to perceived utility or provider comfort, neuromonitoring was employed frequently at our institution among varied types of patients and ages, despite a lack of consensus in the field. We demonstrate high rates of neurological technology use even in patients without primary neurological diseases, with almost one-fourth of patients who received neuro-imaging or neuromonitoring carrying a primary non-neurological diagnosis. Certainly, the non-neurologic populations receiving the most frequent neuromonitoring (respiratory, heme/onc, GI, and infectious/inflammatory diseases) have presumable physiologic drivers prompting the decision to apply neuromonitoring. Secondary neurological insults should be recognized as known risks for many of these critical illnesses. Patients placed on ECMO face the risk of both ischemic and hemorrhagic stroke, as well as the risk for neurologic sequelae of severe respiratory failure or multi-organ system failure that necessitated ECMO support [13,20]. Oncologic malignancies increase risks for stroke, medication-induced encephalopathy, or intracranial hemorrhage due to coagulopathy [21]. Patients with liver failure may require neuromonitoring for hepatic encephalopathy [22]. Neuromonitoring use in these circumstances, determined here at the discretion of the provider or nurse, indicates an awareness and concern for neurologic injury in these disease processes, yet these patients were cared for outside of the neurocritical care settings or protocols. Identifying patient populations which would benefit from routine neuromonitoring, either through evidence or consensus, can help begin the creation of standardized guidance in these at-risk patient populations.

Our study also highlights the need to answer when and how neuromonitoring should be applied. Adjusting for severity of illness, we found that pupillometry use was associated with in-hospital mortality, while the use of other neuromonitoring modalities was associated with new significant disability. Though our data does not purport causality between neuromonitoring use and unfavorable outcomes, these associations may suggest that the use of neuromonitoring in an ad hoc fashion (perhaps as a decision driven by a patient having already clinically deteriorated) is likely not optimal. That is, it may be too late to apply neuromonitoring only after a patient is already severely ill. While neuromonitoring is often used for outcome prediction and prognostication, there is evidence that it can be used more proactively for early detection and prevention of neurological decline [23,24,25]. We did not explore clinical findings in relation to specific neuromonitoring thresholds in this study. Further research should focus on these thresholds in the clinical setting, to not only contribute to our understanding of pathophysiologic trends but to also help establish goal-directed treatment targets that are lacking in children. Some progress has been made in understanding how neuromonitoring can be applied and interpreted for effective clinical use in retrospective studies. Cerebral NIRS, for example, can be used to derive optimal blood pressure goals to target after cardiac arrest in children [17]. Multimodal monitoring can provide cerebral autoregulation and autonomic function indices that may be associated with outcome after pediatric cerebral arteriovenous malformation rupture [18]. After these physiologic thresholds are proposed, prospective studies are needed to determine the effect of neuromonitoring-guided care on outcomes in children.

Retrospective in nature, our study has limitations. It should be noted that data were dependent on assigned diagnoses and documentation in the electronic health record. Broad diagnostic groups encompass wide ranges of specific, and sometimes overlapping, morbidities and are therefore limited in generalizability. As the purpose of this study was to explore associations and not create a predictive model, multicollinearity between variables was not examined. A low frequency of outcomes for certain neuromonitoring modalities, such as TCD and PbtO_2_, did not allow for inclusion in the regression analysis. Detailed data regarding timing, clinical reasoning, and diagnostic subgroups were also not examined, but they would be useful questions for future research in larger cohorts or multicenter studies. Our definition of disability was broad, and other granular measures of disability, such as the pediatric cerebral performance scale or functional status scale, may also be useful, though carrying their own limitations, for outcomes-focused research [26].

## 5. Conclusions

Neuromonitoring is used in diverse patient populations within pediatric critical care, despite the lack of a protocol and consensus. To our knowledge, our results are the first non-survey-based report exploring existing practice tendencies for a vast array of different neuromonitoring modalities in the general pediatric critical care setting. We reveal a recognition around the need to apply neuromonitoring in critically ill children but an association with death and disability when applied in an ad hoc fashion. As such, we suggest that wider and more standardized use of neuromonitoring may be advantageous for earlier intervention, though studies designed to investigate this question specifically are needed. Our data also emphasize the need for further research with the aim to create guidance around the use of neuromonitoring technologies. This guidance should include the selection of patient populations for neuromonitoring, optimal timing of employment, and diagnosis- and age-based thresholds for treatment targets. Advancing our understanding and utilization of neuromonitoring in children is a noteworthy and necessary step in our field if we are to optimize resource allocation, maintain high sensitivity to neurologic injury in critically ill children, and improve outcomes.

## Figures and Tables

**Table 1:** Patient characteristics, illness severity, and outcomes.

Patient Characteristics	All Patients	Primary Neuro Diagnosis	Primary Non-Neuro Diagnosis	*p*
n	1946	542 (27.9%)	1404 (72.1%)	
Female (%)	871 (44.8%)	251 (46.3%)	620 (44.2%)	0.392
Mean age (years) ± SD	7.6 ± 6.4	8.3 ± 6.2	7.4 ± 6.5	<0.001
Mean PRISM III POD (%) ± SD	1.7 ± 6.9	2.1 ± 10.1	1.6 ± 5.2	<0.001
Mean hospital LOS (days) ± SD	11.9 ± 29.1	8 ± 16.7	13.4 ± 32.6	0.23
ECMO	19 (1%)	4 (0.7%)	15 (1.1%)	0.506
New significant disability (%)	98 (5.1%)	38 (7%)	60 (4.3%)	0.013
Deceased (%)	52 (2.7%)	21 (3.9%)	31 (2.2%)	0.041

PRISM III POD = Probability of death as predicted by the highest PICU admission PRISM III score. LOS = Length of stay. New significant disability = new tracheostomy or enteral feeding tube requirement or first-time discharge to inpatient rehabilitation or skilled nursing facility.

**Table 2:** Univariate Associations with Hospital Outcome.

Variable	Deceased (52/1946)		*p*	New Disability (98/1894)		*p*
Age, mean ± SD	8.5 ± 7.5		0.649	6.2 ± 6.2		0.030
PRISM III POD, mean ± SD	18.9 ± 28.2		<0.001	3 ± 7.6		<0.001
	n	OR (95% CI)		n	OR (95% CI)	
Neuro dx	21/542	1.8 (1–3)	0.041	38/521	1.7 (1.1–2.6)	0.010
Respiratory dx	11/804	0.4 (0.2–0.7)	0.003	31/793	0.6 (0.4–1)	0.035
Inflammatory dx	3/118	0.9 (0.3–3)	0.928	3/115	0.5 (0.1–1.5)	0.200
Toxic/Metabolic dx	1/118	0.3 (0–2.2)	0.205	1/117	0.1 (0–1.1)	0.029
GI dx	6/117	2 (0.9–4.7)	0.089	10/111	1.9 (1–3.8)	0.06
Heme/Onc dx	6/81	3 (1.3–6.8)	0.007	7/75	2 (0.9–4.4)	0.097
Other dx	0/68	1 (1–1)	0.164	0/68	1 (1–1)	0.050
Renal dx	1/55	0.7 (0.1–4.8)	0.690	4/54	1.5 (0.5–4.2)	0.452
Cardiovascular dx	3/43	2.7 (0.9–8.4)	0.077	4/40	2.1 (0.7–6)	0.164
ECMO	11/19	27.2 (16.7–44.4)	<0.001	2/8	6.2 (1.2–31.2)	0.011
Neuro-imaging	28/341	5.5 (3.2–9.4)	<0.001	41/313	4 (2.6–6.1)	<0.001
EEG use	27/133	13.6 (8.2–22.8)	<0.001	27/106	8.2 (5–13.4)	<0.001
Automated pupillometry	34/99	35.2 (20.7–60.1)	<0.001	18/65	8.4 (4.7–15.1)	<0.001
NIRS	23/73	20.3 (12.4–33.4)	<0.001	22/50	18.3 (10–33.4)	<0.001
ICP monitoring	3/48	2.4 (0.8–7.5)	0.120	12/45	7.5 (3.7–14.9)	<0.001
TCD	1/5	7.6 (1.3–44.9)	0.016	3/4	56.7 (5.8–550.1)	<0.001
PbtO2 monitoring	0/1	nc	nc	1/1	nc	nc

OR = odds ratio. New disability = new tracheostomy, new feeding tube requirement, new discharge to skilled nursing facility, or new discharge to inpatient rehabilitation among PICU survivors. nc = not calculated. Dx = diagnosis.

**Table 3:** Logistic Regression with Neuromonitoring Use, Covariates, and Hospital Outcome.

Variable	Deceased		New Disability	
	OR (95% CI)	*p*	OR (95% CI)	*p*
Age			1 (0.9–1)	0.034
PRISM III POD	1 (1–1.1)	0.005	1 (0.9–1)	0.222
ECMO	3.5 (0.8–14.7)	0.089		
Neuro Dx	0.8 (0.3–2)	0.652	0.7 (0.4–1.4)	0.372
Neuro-imaging	0.7 (0.2–2.2)	0.571	2.5 (1.2–5.1)	0.012
EEG	1.5 (0.5–4.2)	0.447	3.1 (1.5–6.3)	0.001
Automated pupillometry	27.3 (10.6–69.9)	<0.001	1.1 (0.5–2.8)	0.797
NIRS	1.4 (0.6–3.7)	0.456	10.5 (4.6–23.9)	<0.001
ICP monitoring			2.4 (1–6.2)	0.059

## Data Availability

The data presented in this study are available in the Supplementary Materials listed above.

## Supplementary Materials

Supporting data can be downloaded at: https://www.mdpi.com/article/10.3390/children9070934/s1.
